# Clinometric Gait Analysis Using Smart Insoles in Patients With Hemiplegia After Stroke: Pilot Study

**DOI:** 10.2196/22208

**Published:** 2020-09-10

**Authors:** Minseok Seo, Myung-Jun Shin, Tae Sung Park, Jong-Hwan Park

**Affiliations:** 1 School of Medicine Pusan National University Busan Republic of Korea; 2 Department of Rehabilitation Medicine School of Medicine Pusan National University Busan Republic of Korea; 3 Biomedical Research Institute Pusan National University Hospital Busan Republic of Korea

**Keywords:** stroke, hemiplegia, gait, smart insole, medical informatics, rehabilitation, observational, wearable, assessment

## Abstract

**Background:**

For effective rehabilitation after stroke, it is essential to conduct an objective assessment of the patient’s functional status. Several stroke severity scales have been used for this purpose, but such scales have various limitations.

**Objective:**

Gait analysis using smart insole technology can be applied continuously, objectively, and quantitatively, thereby overcoming the shortcomings of other assessment tools.

**Methods:**

To confirm the reliability of gait analysis using smart insole technology, normal healthy controls wore insoles in their shoes during the Timed Up and Go (TUG) test. The gait parameters were compared with the manually collected data. To determine the gait characteristics of patients with hemiplegia due to stroke, they were asked to wear insoles and take the TUG test; gait parameters were calculated and compared with those of control subjects. To investigate whether the gait analysis accurately reflected the patients’ clinical condition, we analyzed the relationships of 22 gait parameters on 4 stroke severity scales.

**Results:**

The smart insole gait parameter data were similar to those calculated manually. Among the 18 gait parameters tested, 14 were significantly effective at distinguishing patients from healthy controls. The smart insole data revealed that the stance duration on both sides was longer in patients than controls, which has proven difficult to show using other methods. Furthermore, the sound side in patients showed a markedly longer stance duration. Regarding swing duration, that of the sound side was shorter in patients than controls, whereas that of the hemiplegic side was longer. We identified 10 significantly correlated gait parameters on the stroke severity scales. Notably, the difference in stance duration between the sound and hemiplegic sides was significantly correlated with the Fugl-Meyer Assessment (FMA) lower extremity score.

**Conclusions:**

This study confirmed the feasibility and applicability of the smart insole as a device to assess the gait of patients with hemiplegia due to stroke. In addition, we demonstrated that the FMA score was significantly correlated with the smart insole data. Providing an environment where stroke patients can easily measure walking ability helps to maintain chronic functions as well as acute rehabilitation.

**Trial Registration:**

UMIN Clinical Trials Registry UMIN000041646, https://upload.umin.ac.jp/cgi-open-bin/ctr_e/ctr_view.cgi?recptno=R000047538

## Introduction

Stroke remains one of the leading causes of disease burden worldwide [1]. Despite efforts to prevent stroke and reduce its impact with early intervention, many people live with persistent, chronic deficits that require significant rehabilitation [2]. The challenge for stroke rehabilitation is to decrease impairments and promote patient activity and participation by optimizing early outcome prognosis and therapeutic care [3].

To ensure effective rehabilitation therapy for a patient diagnosed with stroke, it is essential to perform an objective assessment of the patient’s functional status. For this purpose, various functional tools have been developed for evaluating patients diagnosed with stroke, such as the Fugl-Meyer Assessment (FMA), the Mini–Mental State Examination (MMSE) for body functions, the Modified Barthel Index (MBI) for activities, and the Stroke Impact Scale for participation, which are the most widely used according to the International Classification of Functioning, Disability, and Health (ICF). However, these scales are time-consuming to administer and problematic due to the influence of the subjective perception of the evaluator [4]. The ordinal scale of these instruments represents a further limitation. Furthermore, it is difficult to record data continuously while administering treatments.

Instrumental gait analysis can be used to evaluate patients continuously, objectively, and quantitatively [5]. Moreover, it can overcome the limitations of other scales, as evaluation and treatment can be simultaneous. We investigated whether in-depth gait analysis using smart insole technology reflects the functional status of patients with hemiplegia due to stroke.

## Methods

### Experimental Design

To investigate the reliability of gait analysis using the smart insole device, healthy control subjects wore the insoles in their shoes and completed a Timed Up and Go (TUG) test. The spatiotemporal gait parameters were compared with the values calculated by manually measuring the TUG time and step count. To determine the gait characteristics of patients with hemiplegia due to stroke, they were asked to wear the insoles and take the TUG. The gait parameters were calculated and compared with those of control subjects. The TUG differentiates subjects with chronic stroke from healthy elderly subjects and shows test-retest reliability [6]. Therefore, this study employed the TUG test for gait analysis of patients diagnosed with stroke. To examine whether the gait analysis data accurately reflected patients’ clinical conditions, we analyzed the relationship between gait parameters and the stroke severity scale data.

### Participants

Participants in this study included 10 healthy control subjects and 10 patients with hemiplegia due to stroke. Included patients were all of chronic-stage status, were diagnosed with stroke, understood the purpose of the experiment, and could walk independently without the use of a walking aid during the TUG test. Patients were excluded if they had musculoskeletal disorders that could affect gait or diabetic complications that could cause peripheral neuropathy. Table 1 displays the characteristics of the 10 patients included in the study.

**Table 1:** Basic characteristics of patients (N=10).

Characteristics	Value, mean (range)
Duration of illness (months)	47.0 (12.0-85.0)
Height (cm)	165.3 (148.0-175.0)
Weight (kg)	69.4 (49.6-97.0)
MMSE^a^	24.4 (14.0-30.0)
MBI^b^	59.0 (22.0-89.0)
FMA_Uex^c^	25.5 (4.0-47.0)
FMA_Lex^d^	19.0 (7.0-28.0)

^a^MMSE: Mini–Mental State Examination.

^b^MBI: Modified Barthel Index.

^c^FMA_Uex: Fugl-Meyer Assessment, upper-extremity score.

^d^FMA_Lex: Fugl-Meyer Assessment, lower-extremity score.

### Measurements

The following functional tools were used to evaluate patients: (1) the Timed Up and Go (TUG) test, (2) the Modified Barthel Index (MBI), (3) the Fugle-Meyer Assessment (FMA), and (4) the Mini–Mental State Examination (MMSE).

#### Timed Up and Go (TUG) Test

**Figure 1:**
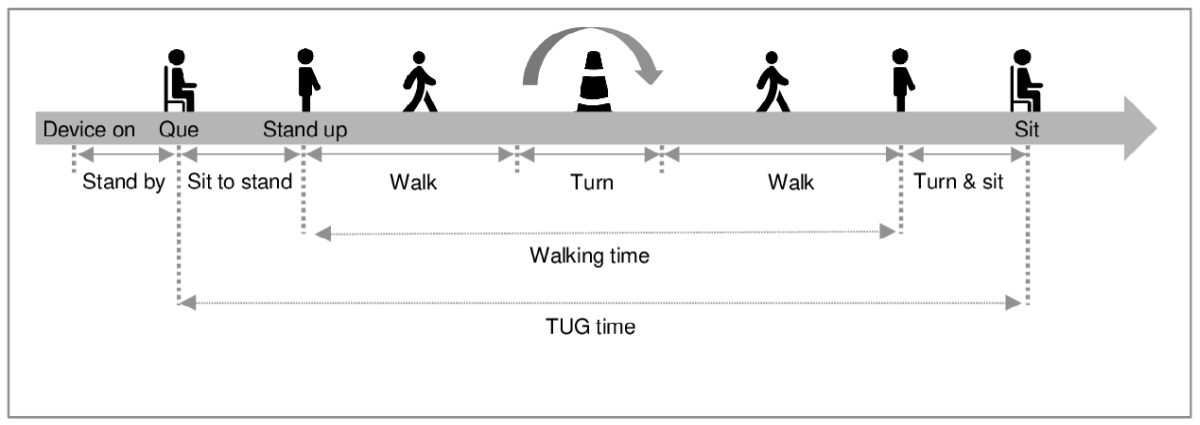
Timed Up and Go (TUG) test.

**Figure 2:**
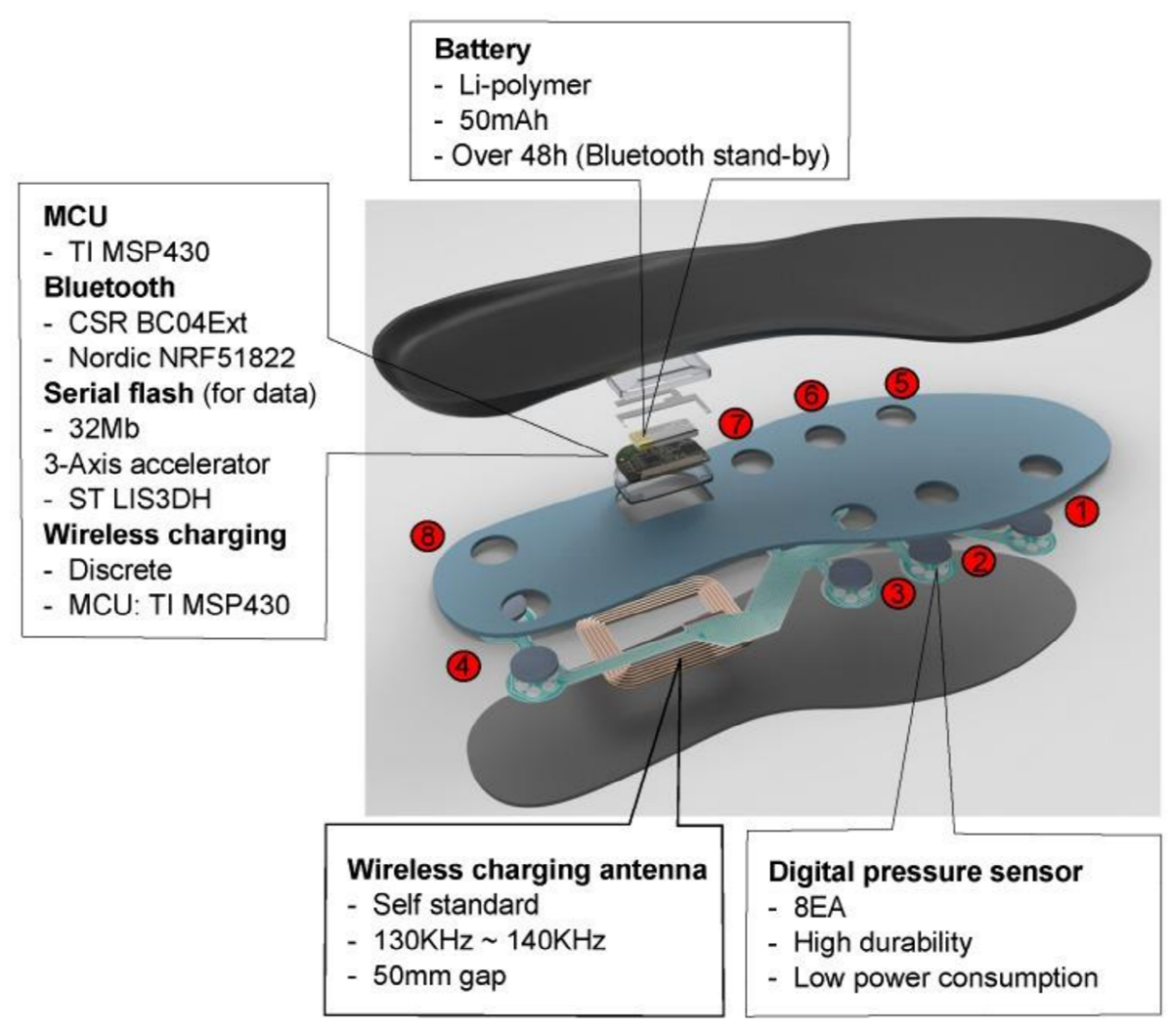
Design of the smart insole sensor module. EA: each; h: hour; kHz: kilohertz; mAh: milliampere hour; Mb: megabytes; MCU: microcontrol unit; mm: millimeter.

The test was performed according to a standard protocol on a standard TUG track (Figure 1) [7]. The patients wore the smart insoles in their shoes. Each insole is equipped with 8 pressure sensors, a 3-axis accelerometer, and a gyroscope; the data were measured at a frequency of 100 Hz (Figure 2).

#### Modified Barthel Index (MBI)

The most widely used tool for evaluating daily activities of stroke patients in the current rehabilitation is the MBI and the functional independence measure (FIM). Because the FIM has restrictions (such as a fee to the copyright holder and the need for training), the MBI is more commonly used in clinical practice and research [8]. The MBI also has a metrological advantage, which is reported to be more sensitive, simpler, easier to score than other tools, more reliable, and more feasible [9,10]. These benefits have contributed to ensuring that the MBI is translated and valid in many countries [8,11-13].

#### Fugl-Meyer Assessment (FMA)

The FMA is a 226-point scale developed to evaluate recovery from hemiplegic stroke. In stroke rehabilitation, it is one of the most comprehensive quantitative measurements of motor impairment (body function). Although the use of the FMA for patients with mild motor impairment is limited by a ceiling effect, the FMA is reliable and is highly recommended as a body impairment scale based on available evidence [14]. Using the FMA in combination with a general activity measure such as the MBI or the TUG may provide additional information to improve the measurement of recovery for stroke patients.

#### Mini–Mental State Examination (MMSE)

The MMSE is frequently used in clinical practice. Although this instrument was originally developed to screen for dementia and delirium, the use of the MMSE has been extended, and many studies now use it as a screening instrument for global cognitive impairment [15]. The Montreal Cognitive Assessment is the best candidate to predict recovery; however, the MMSE is still a useful scale in clinical settings of stroke rehabilitation. As cognitive dysfunction affects learning and rehabilitation outcomes, as well as predicting functional independence after stroke, assessment of cognitive function must also be considered to evaluate the severity of stroke [16].

### Data Handling and Analyses

**Figure 3:**
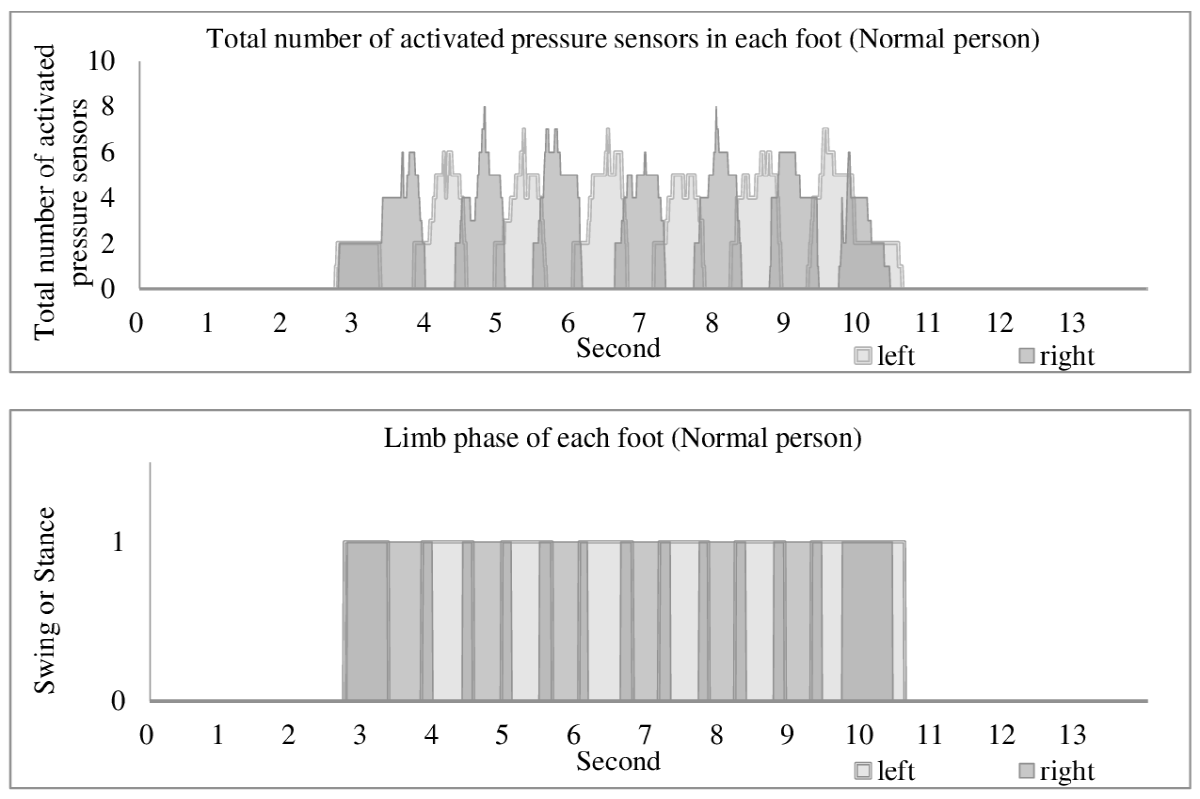
Dividing the swing and stance phase. If the individual press sensor has a pressure of 4.3 N/cm2 or more, it is defined as activated pressure sensor. The swing phase corresponds to when the total number of activated pressure sensors is 0; the stance is represented by non-zero values. If both left and right sides are in stance state, it is treated as double support; if only one side is in stance state, it is treated as single support.

During the experiment, the sensor data were stored in the flash memory in the insole and transmitted via Bluetooth after the experiment. After collecting the data, we performed noise filtering and differentiated between the swing and stance phases by reference to the total number of activated pressure sensors. As shown in Figure 3, the swing phase corresponded to when the total number of activated pressure sensors was 0. The stance was represented by non-zero values, as was described previously by Truong et al [17], who used the same equipment and concluded that their experimental tests performed accurately to distinguish between swing and stance phases. We calculated the gait parameters for each participant, as shown in Table 2. The single support time is when only one foot is in the stance phase, and the double support time is when both feet are in the stance phase. The percentages of difference between swing and stance durations were calculated by dividing the differences in swing and stance durations (measured in seconds) by the corresponding gait cycle duration (measured in seconds). A 2-sample *t* test was used to compare the gait parameters between patients with hemiplegia and normal control subjects, and the coefficient of determination (*R*^2^) was used to analyze the Pearson correlation between gait parameters and stroke severity scale results. The calculations were performed using R statistical software [18].

**Table 2:** Comparison of gait parameters between patients with hemiplegia (n=10) and normal control subjects (n=10), using a 2-sample *t* test.

Gait parameters	Hemiplegia, mean (range)	Normal control, mean (range)	*P* value
Walking speed (meters/second)	0.32 (0.20-0.48)	0.88 (0.73-1.02)	<.001
Stride length (meters)	0.40 (0.23-0.57)	0.91 (0.80-1.00)	<.001
TUG time (seconds)	24.69 (15.92-36.70)	9.83 (8.62-11.26)	<.001
Walking time (seconds)	20.63 (12.48-30.47)	6.95 (5.91-8.24)	<.001
Single support time, SD (%)	0.14 (0.08,0.23)	0.04 (0.02,0.06)	<.001
Single support time, SD (seconds)	0.19 (0.08-0.36)	0.04 (0.03-0.07)	.001
Double support time, mean (seconds)	0.19 (0.13-0.25)	0.13 (0.10-0.16)	.002
Difference in swing duration (%)	0.18 (0.01-0.46)	0.02 (0.00-0.04)	.005
Difference in stance duration (%)	0.19 (0.00-0.45)	0.03 (0.01-0.06)	.005
Difference in stance duration (seconds)	0.27 (0.00-0.56)	0.03 (0.01-0.06)	.005
Difference in swing duration (seconds)	0.25 (0.01-0.54)	0.02 (0.00-0.04)	.006
Double support time, mean (%)	0.15 (0.10-0.21)	0.12 (0.10-0.13)	.03
Sit to standing (seconds)	4.06 (1.19-6.23)	2.88 (2.36-3.62)	.03
Cadence (steps/min)	97.25 (66.64-138.09)	114.49 (97.50-124.60)	.048
Double support time, SD (s)	0.08 (0.02-0.19)	0.05 (0.03-0.05)	.053
Single support time, mean (%)	0.35 (0.30-0.41)	0.38 (0.35-0.41)	.08
Double support time, SD (%)	0.06 (0.02-0.17)	0.04 (0.03-0.05)	.17
Single support time, mean (seconds)	0.47 (0.30-0.66)	0.40 (0.36-0.46)	.19

### Ethics Approval and Consent to Participate

The Institutional Review Board of Pusan National University Hospital approved this study, which was registered retrospectively (Pusan National University Hospital, https://www.pnuh.or.kr; 1812-010-074). This study was registered in the UMIN Clinical Trials Registry (University hospital Medical Information Network, https://www.umin.ac.jp/ctr/; No. UMIN000041646).

## Results

### Control Group

The smart insole results for the control subjects were as follows: TUG time = 8.62–11.26 seconds, cadence = 97.5–124.6 steps/min, walking speed = 0.73–1.02 meters/second, and stride length = 0.80–1.00 meters.

### Patients Diagnosed With Stroke

**Figure 4:**
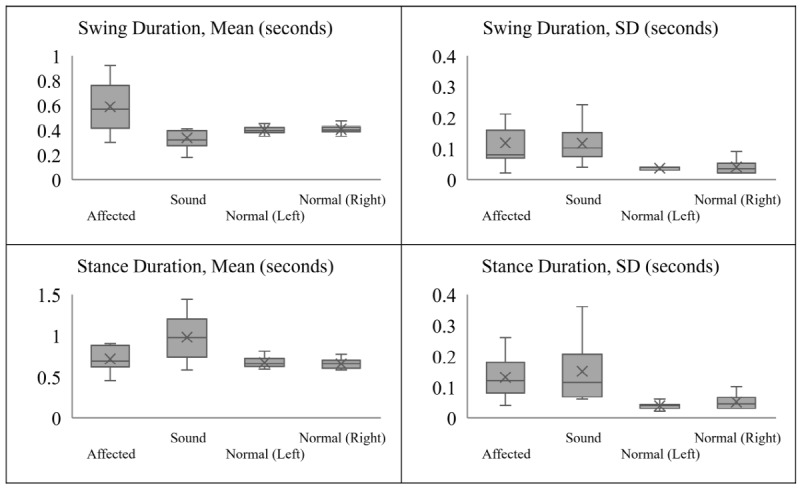
Boxplot displaying the swing and stance duration distribution of patients’ hemiplegic and sound sides, based on a 5-number summary: minimum, maximum, median, first quartile, and third quartile. X: mean. The patients showed a shorter swing duration on the sound side than the control subjects, whereas that on the hemiplegic side was longer. Patients showed a longer stance duration on both sides compared to the control subjects; however, the sound side showed a markedly longer duration.

The patients diagnosed with stroke had a mean duration of disease of 47.0 (SD 29.4) months and a mean MMSE score of 24.4 (SD 4.9). The gait parameters that showed significant differences between patients with hemiplegia and normal control subjects were the TUG time (seconds), walking time (seconds), sit-to-standing time (seconds), cadence (steps/min), walking speed (meters/second), stride length (meters), standard deviation of single support time (seconds, percentage), mean double support time (seconds, percentage), difference in swing duration between the sound and hemiplegic sides (seconds, percentage) and difference in stance duration between the sound and hemiplegic sides (seconds, percentage) (Table 2). The patients had a mean TUG time of 24.69 seconds, which was longer than that of the control subjects (9.83 seconds). The walking speed range of the patients with hemiplegia was 0.20 to 0.48 meters per second, which was significantly slower than that of control subjects (0.73-1.02 meters per second). The cadence of the patients was 66.64-138.09 steps per minute, which was more broadly distributed than that of the control subjects (97.50-124.60 steps per min), and their stride length was 0.23-0.57 meters, which was significantly shorter than that of control subjects (0.80-1.00 meters). The results showed that the patients had a slower walking speed due to shorter stride length and higher cadence. The average single support time was 0.47 seconds (mean value of the hemiplegic and sound sides), which did not differ significantly from that of control subjects (0.40 seconds). However, the mean difference in stance duration between the sound and hemiplegic sides was 0.27 seconds, which was significantly greater than that of control subjects (0.03 seconds) (Figure 4).

### Correlations Between Gait Parameters and Stroke Severity Scale Results

**Figure 5:**
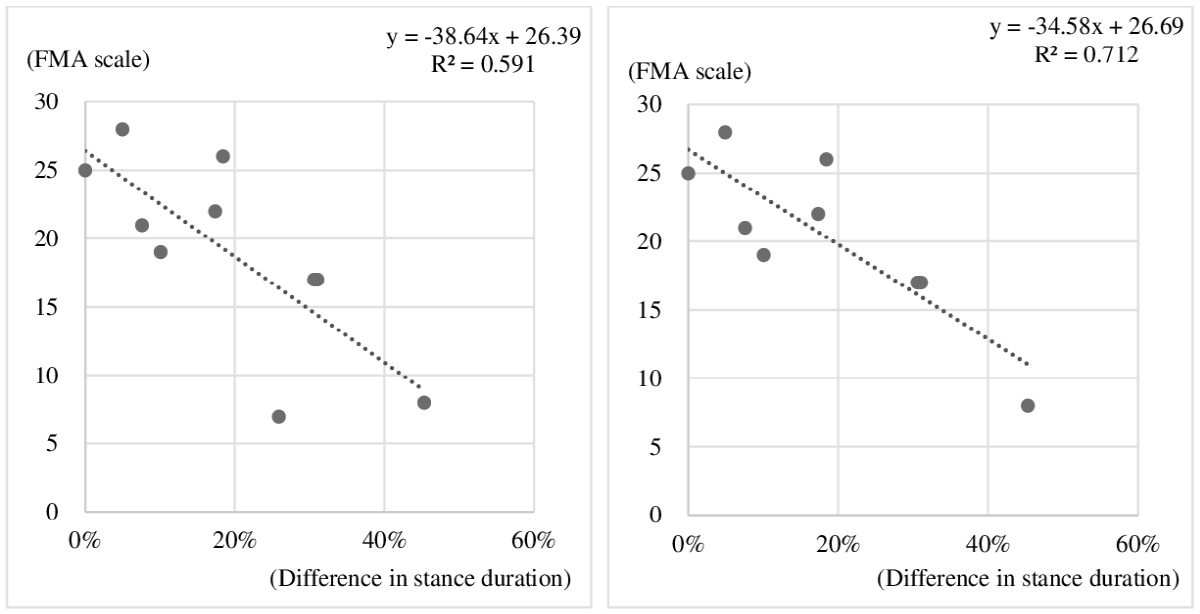
Correlation between the difference in stance duration and the Fugl-Meyer Assessment (FMA) lower-extremity score. The recalculated R² value, excluding an outlier patient, was larger at 0.712.

As shown in Table 3 and Figure 5, the difference in stance duration between the sound and hemiplegic sides was most strongly correlated with the FMA lower extremity score in the hemiplegia group (y = -38.64x + 26.39, R² = 0.59). The recalculated R² value, excluding an outlier patient, was larger at 0.71, indicating a clear correlation between the FMA lower extremity score and the difference in stance duration between the sound and hemiplegic sides.

The 4 stroke severity scales used for correlation analysis were the FMA for the lower extremity and the upper extremity, the MMSE, and the MBI. The 22 gait parameters used for correlation analysis were the TUG time (seconds), walking time (seconds), sit-to-standing time (seconds), cadence (steps per minute), walking speed (meters per second), stride length (meters), mean single support time (seconds), standard deviation of single support time (seconds), mean double support time (seconds), standard deviation of double support time (seconds), difference in swing duration (seconds), difference in stance duration (seconds), percentage difference in swing duration, percentage difference in stance duration, mean swing duration of hemiplegic side (seconds), standard deviation of swing duration of hemiplegic side (seconds), mean swing duration of sound side (seconds), standard deviation of swing duration of sound side (seconds), mean stance duration of hemiplegic side (seconds), standard deviation of stance duration of hemiplegic side (seconds), mean sound-side stance duration (seconds), and standard deviation of sound-side stance duration (seconds).

**Table 3:** Correlations between gait parameters and stroke severity scales.

Gait parameter	Stroke severity scale	R^2^	*P* value^a^
Difference in stance duration (%)	FMA_Lex^b^	0.591	.009
Difference in stance duration (%)	FMA_Uex^c^	0.539	.02
Difference in swing duration (%)	FMA_Lex^b^	0.517	.02
Sound-side swing duration, SD (seconds)	MMSE^d^	0.498	.02
Cadence (steps/min)	MMSE^d^	0.484	.03
Hemiplegic-side stance duration, SD (seconds)	MMSE^d^	0.452	.03
Single support time, mean (seconds)	MMSE^d^	0.448	.03
Sound-side stance duration, mean (seconds)	MMSE^d^	0.442	.04
Walking speed (meters/second)	MMSE^d^	0.403	.048
Single support time, SD (seconds)	MMSE^d^	0.401	.049

^a^Non-significant results omitted.

^b^FMA_Lex: Fugl-Meyer Assessment, lower-extremity score.

^c^FMA_Uex: Fugl-Meyer Assessment, upper-extremity score.

^d^MMSE: Mini–Mental State Examination.

## Discussion

### Feasibility and Applicability of the Smart Insole as a Gait Analysis Device

As a gait analysis device, the smart insole showed that it may be used in place of the manual calculations of gait parameters made by a clinician, such as calculations of TUG time and step count. Truong et al [17] concluded that this equipment made accurate estimates of walking distance with a mean walking distance estimation error of 4.8% and 3.1% for 16 meters and 89 meters walking distance, respectively. David et al [19] demonstrated the feasibility and applicability of rehabilitation using an eSHOE system similar to the smart insole device. The smart insole used in this study was also applied to predict energy consumption in a recent article [20]. Thus, we suggest that these types of systems are more useful than other methods as they are location-independent and can measure additional parameters that have not been addressed in the past.

### Gait Features of Patients With Hemiplegia due to Stroke

**Figure 6:**
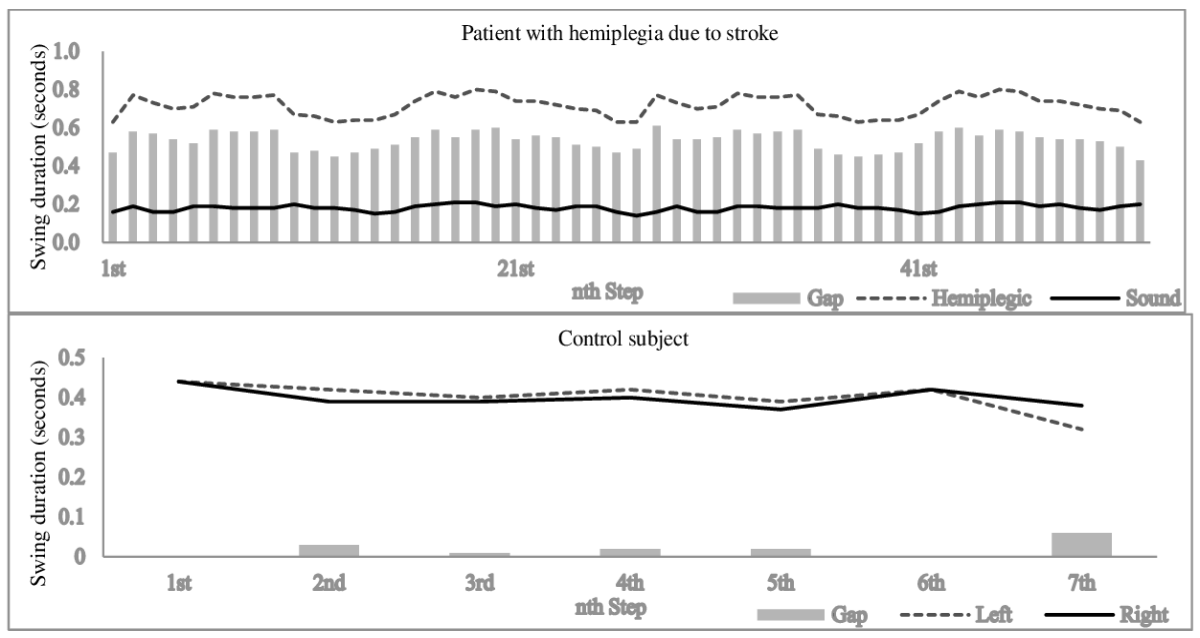
Difference in swing duration between a patient with hemiplegia and a normal control subject. The control subject showed a difference close to zero (ie, the left- and right-sided swing durations were almost the same); however, the patient showed a longer swing duration on the hemiplegic side than the sound side, with the difference exceeding 0.4 seconds.

Analysis of the smart insole data revealed several gait characteristics of patients with hemiplegia due to stroke that were difficult to identify using previous methods. First, the patients showed slower walking speeds and shorter stride lengths than the control subjects. To compensate for these differences, some patients showed a faster cadence than control subjects. As shown in Figure 4, we observed a difference between the hemiplegic and sound sides. While the patients showed a longer stance duration on both sides compared to control subjects, the sound side showed a markedly longer duration. In addition, the patients showed a shorter swing duration on the sound side than the control subjects, whereas that on the hemiplegic side was longer. It is likely that the hemiplegic side moved slowly during the swing phase and lacked the strength required to support the body’s weight during the stance phase. Figure 6 shows the difference in swing duration between the sound and hemiplegic sides for a patient and between the left and right sides for a control subject. The control subject showed a difference close to zero (ie, the left- and right-sided swing durations were almost the same). However, the patient showed a longer swing duration on the hemiplegic side than the sound side, with the difference exceeding 0.4 seconds. In an analysis of the relationship between TUG performance and gait parameters, Bonnyaud et al [21] revealed the importance of swing and stance duration, where the motor ability of the paretic lower limb and the single support phase on the paretic side determined TUG performance. The results obtained with the smart insole were consistent with the previous study and could be obtained more easily.

### Gait Analysis for Clinical Assessment

We examined the correlations of 22 gait parameters with stroke severity scale data, such as FMA lower- and upper- extremity scores, and MMSE and MBI scores. Of the 22 gait parameters, 10 showed significant correlations (Table 3). The strongest correlations were found between the FMA lower-extremity score and the difference in stance duration between the sound and hemiplegic sides.

As the MMSE is used for measuring cognitive impairment, it seems that it has an overall correlation with several gait parameters rather than having a high correlation with specific gait parameters. In this study, the MMSE does not have a high correlation with a specific gait parameter like the FMA lower extremity score does. However, the MMSE has a significant correlation with most types (7) of gait parameters (Table 3). We expect that the variability of gait parameters could be related to cognitive impairment due to a decrease in the efficiency of rehabilitation training. In this study, we found that patients diagnosed with stroke have a greater standard deviation for gait parameters, which is more meaningful than the average (Table 2, Figure 4). Thus, if there were patients with the same motor impairment but with more severe cognitive impairments, they would possibly have been distinguished by the variability of gait parameters during TUG, a complex task.

MBI does not have a clear correlation with specific gait parameters because it evaluates the performance of daily life activities, and not just walking ability. In contrast, the FMA is an instrument for measuring recovery from sensorimotor stroke [22], and it can evaluate various body parts and functions. Thus, the FMA should intuitively have a direct connection with walking ability.

Guzika et al [23] emphasized the importance of gait symmetry, for which only parameters measuring gait symmetry were associated with the degree of gait control in patients after stroke diagnosis. In this respect, it is reasonable that differences in stance duration and swing duration between the sound and hemiplegic sides would show the most significant correlations with FMA lower-extremity scores, which reflect gait ability.

In addition, Hiengkaew et al [24] reported that both the FMA lower-extremity score and TUG were reliable measurements of postural balance and lower limb movements in individuals with chronic stroke. As the stability of the swing and stance phases determine TUG performance [21], and the FMA lower-extremity score has a significant correlation with the difference in swing and stance duration, a correlation between FMA lower-extremity score and TUG could be deduced. No direct correlation was found between TUG time and the FMA lower-extremity score in this experiment; however, it was found that gait parameters during TUG had a significant correlation with the FMA lower-extremity score (Table 3). As the TUG includes a return section, it is more complex than the walking test for straight distances and can reflect the problem of hemiplegic impairment well. Thus, despite the small number of subjects in this study, the gait parameters during TUG appear to have a significant correlation with the FMA lower-extremity scores. However, if we apply TUG in a conventional way, time information would have been the only information we could have.

We further investigated the outlier patient (Figure 5), who had chronic hemiplegia for 6 years. Although the patient had a relatively low FMA score, the difference in swing duration between the sound and hemiplegic sides was not significant. While the TUG time and cadence of the patient were average for the patient group, the prolonged mean support time suggested instability. Although there were no specific symptoms or signs at the time of the experiment, the patient had a history of treatments for poststroke seizure, which was the only difference from other patients. However, it was difficult to explain the clear reason. We compared the FMA and gait analysis results of the patient with medical records held by other rehabilitation specialists and noted differences in opinion indicating underestimation of the FMA results relative to function. Despite the small number of subjects, the reason for accounting for the outlier is not to assert a clear correlation but rather to show potential for a new method. Future studies with larger numbers of patients are important for further analysis of the characteristics of such outlier patients.

### Future Clinical Applications

Yu et al [25] developed an ensemble model that can estimate the 33-item FMA upper-extremity score based on measured values using wearable devices. Gait is more difficult to analyze as it requires more complex interactions of nerves and muscles; however, it seems to be possible to estimate each item of existing functional evaluations through in-depth gait analysis in follow-up studies.

Gait analysis using the smart insole can be performed without temporal or geographical constraints and might be useful for promoting appropriate gait patterns. This study showed that the smart insole measuring device could replace existing functional evaluations such as the FMA. The smart insole could be used for biofeedback training, both at home and in the hospital environment. Providing an environment where stroke patients can easily measure walking ability may help to maintain chronic functions as well as acute rehabilitation.

### Conclusions

We introduce the feasibility and utility of the smart insole for assessing gait features in patients diagnosed with hemiplegia. In addition, we found that the results for the FMA functional index, which is the most commonly used instrument for assessing patients with motor impairment, were significantly correlated with those obtained using the smart insole. Further studies are required to confirm the clinical effectiveness of the smart insole for rehabilitation treatment and long-term monitoring of patients after a stroke diagnosis.

## Abbreviations

FIM: functional independence measure
FMA: Fugl-Meyer Assessment
MBI: Modified Barthel Index
MMSE: Mini-Mental State Examination
TUG: Timed Up and Go

## References

[1] Feigin VL, Krishnamurthi RV, Parmar P, Norrving B, Mensah GA, Bennett DA, Barker-Collo S, Moran AE, Sacco RL, Truelsen T, Davis S, Pandian JD, Naghavi M, Forouzanfar MH, Nguyen G, Johnson CO, Vos T, Meretoja A, Murray CJ, Roth GA (2015). Update on the Global Burden of Ischemic and Hemorrhagic Stroke in 1990-2013: The GBD 2013 Study. Neuroepidemiology.

[2] Benjamin E, Virani S, Callaway C, Chamberlain A, Chang A, Cheng S, Chiuve Stephanie E, Cushman Mary, Delling Francesca N, Deo Rajat, de Ferranti Sarah D, Ferguson Jane F, Fornage Myriam, Gillespie Cathleen, Isasi Carmen R, Jiménez Monik C, Jordan Lori Chaffin, Judd Suzanne E, Lackland Daniel, Lichtman Judith H, Lisabeth Lynda, Liu Simin, Longenecker Chris T, Lutsey Pamela L, Mackey Jason S, Matchar David B, Matsushita Kunihiro, Mussolino Michael E, Nasir Khurram, O'Flaherty Martin, Palaniappan Latha P, Pandey Ambarish, Pandey Dilip K, Reeves Mathew J, Ritchey Matthew D, Rodriguez Carlos J, Roth Gregory A, Rosamond Wayne D, Sampson Uchechukwu K A, Satou Gary M, Shah Svati H, Spartano Nicole L, Tirschwell David L, Tsao Connie W, Voeks Jenifer H, Willey Joshua Z, Wilkins John T, Wu Jason Hy, Alger Heather M, Wong Sally S, Muntner Paul, American Heart Association Council on EpidemiologyPrevention Statistics CommitteeStroke Statistics Subcommittee (2018). Heart Disease and Stroke Statistics-2018 Update: A Report From the American Heart Association. Circulation.

[3] Bernhardt J, Hayward KS, Kwakkel G, Ward NS, Wolf SL, Borschmann K, Krakauer JW, Boyd LA, Carmichael ST, Corbett D, Cramer SC (2017). Agreed definitions and a shared vision for new standards in stroke recovery research: The Stroke Recovery and Rehabilitation Roundtable taskforce. International Journal of Stroke.

[4] Hamacher D, Singh N, Van Dieën J, Heller M, Taylor W (2011). Kinematic measures for assessing gait stability in elderly individuals: a systematic review. J. R. Soc. Interface.

[5] Tamburini P, Mazzoli D, Stagni R (2018). Towards an objective assessment of motor function in sub-acute stroke patients: Relationship between clinical rating scales and instrumental gait stability indexes. Gait & Posture.

[6] Ng SS, Hui-Chan CW (2005). The Timed Up & Go Test: Its Reliability and Association With Lower-Limb Impairments and Locomotor Capacities in People With Chronic Stroke. Archives of Physical Medicine and Rehabilitation.

[7] Podsiadlo D, Richardson S (1991). The timed. J Am Geriatr Soc.

[8] Jung HY, Park BK, Shin HS, Kang YK, Pyun SB, Paik NJ (2007). Development of the Korean Version of Modified Barthel Index (K-MBI): Multi-center Study for Subjects with Stroke. Journal of the Korean Academy of Rehabilitation Medicine.

[9] Shah S, Vanclay F, Cooper B (1989). Improving the sensitivity of the Barthel Index for stroke rehabilitation. Journal of Clinical Epidemiology.

[10] Duffy L, Gajree S, Langhorne P, Stott DJ, Quinn TJ (2013). Reliability (Inter-rater Agreement) of the Barthel Index for Assessment of Stroke Survivors. Stroke.

[11] Leung SO, Chan CC, Shah S (2016). Development of a Chinese version of the Modified Barthel Index — validity and reliability. Clin Rehabil.

[12] Küçükdeveci AA, Yavuzer G, Tennant A, Süldür N, Sonel B, Arasil T (2000). Adaptation of the modified Barthel Index for use in physical medicine and rehabilitation in Turkey. Scand J Rehabil Med.

[13] Shah S, Cooper B, Maas F (1992). The Barthel Index and ADL Evaluation in Stroke Rehabilitation in Australia, Japan, the UK and the USA. Aust Occup Ther J.

[14] Gladstone DJ, Danells CJ, Black SE (2016). The Fugl-Meyer Assessment of Motor Recovery after Stroke: A Critical Review of Its Measurement Properties. Neurorehabil Neural Repair.

[15] Nys G, Vanzandvoort M, Dekort P, Jansen B, Kappelle L, Dehaan E (2005). Restrictions of the Mini-Mental State Examination in acute stroke. Archives of Clinical Neuropsychology.

[16] Mullick AA, Subramanian SK, Levin MF (2015). Emerging evidence of the association between cognitive deficits and arm motor recovery after stroke: A meta-analysis. RNN.

[17] Truong P, Lee J, Kwon A, Jeong G (2016). Stride Counting in Human Walking and Walking Distance Estimation Using Insole Sensors. Sensors.

[18] www.R-project.org.

[19] David V, Forjan M, Martinek J, Kotzian S, Jagos H, Rafolt D (2017). Evaluating wearable multimodal sensor insoles for motion-pattern measurements in stroke rehabilitation - A pilot study. IEEE Int Conf Rehabil Robot.

[20] Kim SH, Kim JW, Park J, Shin MJ, Choi M (2019). Predicting Energy Expenditure During Gradient Walking With a Foot Monitoring Device: Model-Based Approach. JMIR Mhealth Uhealth.

[21] Bonnyaud C, Pradon D, Zory R, Bensmail D, Vuillerme N, Roche N (2015). Gait parameters predicted by Timed Up and Go performance in stroke patients. NRE.

[22] Gladstone DJ, Danells CJ, Black SE (2002). The fugl-meyer assessment of motor recovery after stroke: a critical review of its measurement properties. Neurorehabil Neural Repair.

[23] Guzik A, Drużbicki M, Przysada G, Kwolek A, Brzozowska-Magoń A, Sobolewski M (2017). Relationships between walking velocity and distance and the symmetry of temporospatial parameters in chronic post-stroke subjects. Acta Bioeng Biomech.

[24] Hiengkaew V, Jitaree K, Chaiyawat P (2012). Minimal Detectable Changes of the Berg Balance Scale, Fugl-Meyer Assessment Scale, Timed “Up & Go” Test, Gait Speeds, and 2-Minute Walk Test in Individuals With Chronic Stroke With Different Degrees of Ankle Plantarflexor Tone. Archives of Physical Medicine and Rehabilitation.

[25] Yu L, Xiong D, Guo L, Wang J (2016). A remote quantitative Fugl-Meyer assessment framework for stroke patients based on wearable sensor networks. Computer Methods and Programs in Biomedicine.

